# Long Complete Remission Achieved with the Combination Therapy of Cisplatin and Gemcitabine in a Patient with Aggressive Natural Killer Cell Leukemia

**DOI:** 10.1155/2015/715615

**Published:** 2015-01-28

**Authors:** Justin LaPorte, Lawrence Morris, John Koepke

**Affiliations:** ^1^The Blood and Marrow Transplant Program at Northside Hospital, Atlanta, GA 30342, USA; ^2^Northside Hospital Pathology Department, Atlanta, GA 30342, USA

## Abstract

Aggressive natural killer cell leukemia (ANKL) is a rare and often lethal lymphoproliferative disorder. Patients may present with constitutional symptoms, jaundice, skin infiltration, lymphadenopathy, and hepatosplenomegaly. ANKL can progress quickly to multiorgan failure and survival is usually measured in weeks. Although a rapid and accurate diagnosis is critical, unfortunately there is no hallmark diagnostic marker of ANKL. We report a case of a 48-year-old male who was able to obtain a complete remission following cisplatin-based chemotherapy. We describe the details of the chemotherapy regimens used and a literature review of the treatment of ANKL.

## 1. Introduction

Lymphoproliferative disorders of natural killer (NK) cells are rare diseases that account for less than 5% of all lymphoid malignancies. ANKL was first described about 30 years ago and is now regarded as a distinct subtype by the World Health Organization [1]. Although ANKL occurs worldwide, it is most prevalent in Asian countries and is characterized by the proliferation of NK-cells that are usually CD3+_c_, CD2+, CD16+, and CD56+ [2]. Morphologically, ANKL can range from large granular lymphocytes to pleomorphic cells having multiple nuclei [3]. ANKL is closely associated with Epstein-Barr virus [4]. Men and women are equally affected and patients are typically in their third to fourth decade of life [5]. Patients with ANKL have a dismal prognosis with a median survival of less than 2 months [3]. Published treatment regimens are limited to case reports and small retrospective cohorts. We report a case of a 48-year-old man with ANKL that achieved a complete remission after cisplatin-based chemotherapy. His disease relapsed before a planned allogeneic hematopoietic stem cell transplant (HSCT).

## 2. Case Presentation

A 48-year-old man was diagnosed with primary myelofibrosis in March 2012 with symptoms of fatigue, fever, splenomegaly, and fibrotic marrow. Polymerase chain reaction (PCR) for a JAK2 mutation was negative. He underwent a splenectomy in July 2012 with no relief in symptoms. He was prescribed prednisone and ruxolitinib which provided relief. Prednisone was eventually tapered off and the symptoms returned. Therefore, ruxolitinib was increased and prednisone was resumed. In March 2013 flow cytometry of peripheral blood revealed atypical intermediate sized mononuclear NK-cells comprising about 77% of the population.

The patient was admitted to the inpatient leukemia service for further evaluation and management. Presenting symptoms included significant malaise, fevers, anorexia, night sweats, and mild dyspnea on exertion. Physical exam was unremarkable. Laboratory studies revealed a white blood cell (WBC) count of 46 K/uL with 75% of the cells representing an NK-cell lymphoproliferative disorder. The patient had slightly elevated glucose, AST/ALT, and LDH. Epstein-Barr virus (EBV) serology was positive; however, the EBV PCR was negative. No disseminated intravascular coagulopathy was present. Computed tomography (CT) scans of the head (including sinuses), chest, abdomen, and pelvis were unremarkable. The bone marrow biopsy with aspiration revealed 30% involvement with small to intermediate sized mononuclear cells, some with inconspicuous nucleoli and small to moderate amounts of cytoplasm. Flow cytometry from the bone marrow biopsy was consistent with the peripheral blood with NK-cells comprising 71% of the population and expressing CD2, CD7, low density CD8, CD16, CD38, CD45, and bright CD56. Given the atypical morphological presentation, clinical correlation was used to determine the diagnosis of ANKL.

After 24 hours of hydroxyurea, induction chemotherapy was initiated which consisted of cyclophosphamide (300 mg/m^2^ every 12 hours D 1–3), vincristine (2 mg D 4 and 11), doxorubicin (50 mg/m^2^ D 4), dexamethasone (40 mg/day D 1–4 and 11–14), and pegaspargase (2,500 units/m^2^ D 4) in combination with intrathecal methotrexate and cytarabine.

Ten days following induction therapy initiation the WBC count had decreased to 2.1 K/uL, but repeat flow cytometry from peripheral blood demonstrated persistent NK-cells involving 70% of the population. The patient developed conjugated hyperbilirubinemia and jaundice. Therefore, salvage chemotherapy was administered consisting of gemcitabine (800 mg/m^2^ (dose adjusted from 1000 mg/m^2^ due to high total bilirubin) D 1, 4, and 8), cisplatin (100 mg/m^2^ D 2), and dexamethasone (20 mg/m^2^ D 1–4). Prophylactic intrathecal treatments were continued.

A second bone marrow biopsy conducted in May 2013 demonstrated no evidence of leukemia on morphology or flow cytometry. The plan was to repeat gemcitabine/cisplatin/dexamethasone every 28 days. The second cycle was delayed by 2 weeks due to grade 3/4 mucositis, malnutrition, elevated liver enzymes, vancomycin-resistant enterococcus bacteremia, and candida parapsilosis fungemia. Cycle 2 was given and the patient was discharged from the hospital. Cycle 3 was delayed because of poor performance status, altered mental status, and CMV viremia requiring ganciclovir treatment. Upon clinical improvement, cycle 3 was given in mid-July 2013. Repeat bone marrow biopsies in June 2013 and September 2013 continued to demonstrate a complete remission.

The patient was then scheduled to undergo HSCT from a matched-unrelated donor; however, the pretransplant bone marrow biopsy revealed new complex cytogenetics. Morphology and flow cytometry remained negative. Decitabine (20 mg/m^2^ D 1–10) was administered. The patient's clinical course was further complicated by dehydration, declining performance status, persistent culture-negative fevers, hypoglycemia, and metabolic acidosis. A bone marrow biopsy done in November 2013 revealed NK-cells comprising 35% of the population. Hospice care was arranged and the patient died in late-November 2013, 8 months after being diagnosed with ANKL.

## 3. Discussion

Currently, there is no optimal chemotherapy regimen to treat ANKL. ANKL is inherently resistant to certain cytotoxic agents through the expression of P-glycoprotein [6]. Therefore, CHOP (cyclophosphamide, doxorubicin, vincristine, and prednisone) based therapy can be ineffective because vincristine and doxorubicin are exported out of the cell by P-glycoprotein [7, 8]. Medications that are not P-glycoprotein substrates like L-asparaginase and methotrexate have demonstrated successful therapeutic results [5, 6].

In a study of 34 ANKL patients conducted by Ishida and colleagues, 5 patients received L-asparaginase containing regimens. Overall survival was extended significantly with L-asparaginase regimens, and, in a univariate analysis, it was the only clinical factor associated with better survival [5]. Based upon this data, our initial choice of therapy was pegaspargase (due to unavailability of L-asparaginase) combined with the Hyper-CVAD induction regimen. Unfortunately, our patient's ANKL was refractory to the pegaspargase-based regimen.

In a study by Zhang and colleagues, 4 patients with relapsed or refractory disease received gemcitabine, cisplatin, and dexamethasone as a salvage regimen. Three of these patients attained a CR for more than 3 months [2]. Gemcitabine has activity in heavily pretreated lymphoma patients. Moreover, gemcitabine and cisplatin are not P-glycoprotein substrates [7]. Due to the evidence of refractory disease, we administered the cisplatin-based regimen, which induced a CR. Larger studies are needed to evaluate the efficacy of this regimen. Although our patient was unable to undergo allogeneic HSCT, this therapeutic option might have a significant role in the treatment of ANKL [5, 6, 9]. Ishida and colleagues demonstrated a median survival of 36 days versus 266 days in patients without HCST compared to those with HSCT, respectively [5].

Interestingly, our patient experienced significant liver dysfunction following a CHOP-like regimen (Hyper-CVAD). This phenomenon was also reported in the study by Zhang and colleagues where 6 of the 12 patients that received CHOP therapy rapidly died because of liver dysfunction and irreversible intravascular coagulation. Liver biopsies from 2 of the patients revealed spreading infiltration of LGLs and patchy hemorrhage [2]. Careful evaluation and potential treatment modification based on organ function are needed before the start of therapy.

Our case demonstrates the aggressiveness characteristics of ANKL. However, complete remission can be achieved in this inherently resistant disease when using medications that are not P-glycoprotein substrates. Although our patient ultimately died with progressive ANKL, achieving a remission may allow some patients to proceed to potentially curative allogeneic HSCT.
